# More than meets the eye: a scoping review on the non-medical uses of THZ eye drops

**DOI:** 10.1007/s12024-023-00680-9

**Published:** 2023-07-28

**Authors:** Esraa Menshawey, Rahma Menshawey

**Affiliations:** Kasr al Ainy Faculty of Medicine, Cairo University, Geziret Elroda, Cairo Manial, 11562 Egypt

**Keywords:** Tetrahydrozoline, Visine, Murder, Rape, Intoxication, Eyedrops, Nasal decongestants, Adulterated drug tests

## Abstract

Tetrahydrozoline is an alpha agonist imidazole derivative found in over-the-counter decongestive eye and nasal drops. The drug was patented in 1954 and was available for medical use in 1959. This drug recently gained the attention of law enforcement as it has been utilized in criminal activity such as homicide and drug-facilitated sexual assault. The aim of this scoping review is to scope the literature for all mentions of tetrahydrozoline eye/nasal drops use in a non-medical context to delineate areas of future research and development. We used Google Scholar and PUBMED/Medline databases to search for non-medicinal and criminal uses of THZ. The search word used was “tetrahydrozoline.” A total of 15 articles matched our criteria. Among the case reports, two (11.1%) cases reported on drug-facilitated sexual assault, and two (11.1%) cases used THZ eyedrops to attempt suicide. Incidental ingestion of THZ eyedrops was reported in eight (44.4%) cases, three (16.7%) cases of attempted murder were reported, two (11.1%) cases of intentional ingestion were reported, and one (5.5%) case was a combination of drug-facilitated sexual assault and attempted murder. The most common clinical presentation was unexplained and resistant bradycardia and hypotension. THZ eye drops can be used to produce false negative results on drug tests. This study recognizes that THZ can be used in non-medicinal and criminal uses. There is room for future research and development. More studies should be conducted to better understand the mechanism of action, therapeutic window, and toxicity levels among various age groups at different methods of intake and to find an effective treatment in case of overdose. Eyedrop and nasal decongestant bottles should be designed with child proofing to prevent incidental ingestion and should contain warning labels. A fast and alternative test to GC/MS can be developed to ease the diagnosis of THZ toxicity. Purchases of this medication may need to be monitored.

## Background

Tetrahydrozoline (THZ) is an alpha agonist imidazole derivative. It is the medicinal ingredient found in several types of over-the-counter eye drops (for conjunctivitis) and nasal decongestants. THZ stimulates the alpha 1 receptor causing local vasoconstriction. Its effects on the alpha 2 receptor (crosses the blood brain barrier) have been reported to reduce central sympathetic outflow causing unopposed parasympathetic activity (bradycardia, hypotension, and CNS depression) [1–3].

Several studies have noted that THZ is rapidly absorbed systemically following topical administration and its effects vary depending on the method of administration. When THZ drops is applied as intended (topically on ocular/ nasal tissue), it is virtually harmless. If THZ is given parentally or enterally, it rapidly distributes within the central nervous system causing inhibition of the sympathetic vasomotor centers as well as reduced peripheral vascular tone. Reported side effects of THZ include dry mouth, miosis, CNS depression, bradycardia, hypotension, respiratory depression, hyporeflexia, and hypothermia [2–6].

Studies have demonstrated that THZ serum/ urine values exceeding the upper limit (13–210 ng/ml in blood or 11–40 ng/ml in urine) is suggestive of non-medicinal use [7]. In addition, recent headlines have been made regarding its use as an agent to facilitate murder, rape, and suicide [5, 8, 9]. Tetrahydrozoline drops are easily accessible and do not arouse suspicion by pharmacists, police investigators, and victims. It is odorless, colorless, and tasteless, and a very minute amount, if ingested, can be fatal.

Unfortunately, there is limited information on THZ pharmacokinetics and pharmacodynamics when ingested orally. Rapid diagnostic methods and treatment modalities for its toxicity are also scarce. The aim of this scoping review is to scope the literature for all mentions of tetrahydrozoline eye/nasal drops use in a non-medical context in order to delineate areas of future research and development.

## Methods

This scoping review was conducted in accordance with the *Joanna Briggs Institute (JBI) Guidelines* for Scoping Reviews.

### Search strategy

**Fig. 1:**
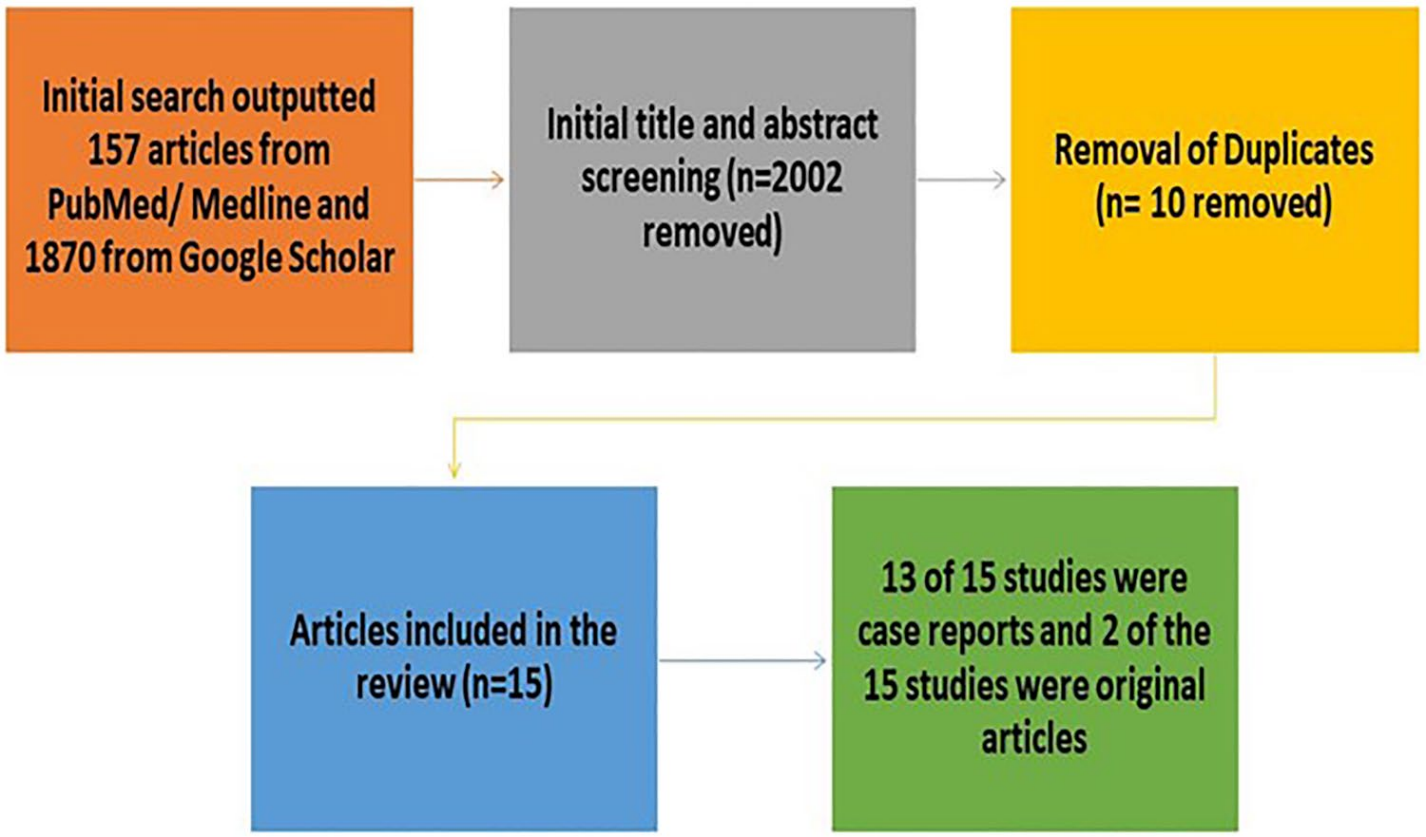
Flowchart for the studies included and excluded

We used Google Scholar and PUBMED/Medline databases to search for non-medicinal and criminal uses of THZ from February 20^th^ 2023. The search word used was “Tetrahydrozoline” (THZ). A total of 157 articles were found using the PUBMED/Medline database and 1870 articles using Google Scholar, with a total of 2027 articles overall. After careful screening of all the articles, a total of 15 studies were included in this scoping review (Fig. 1).

### Inclusion criteria

All English-only full articles on non-medicinal criminal uses of tetrahydrozoline (THZ) eye/nasal drops in the form of adult/ pediatric overdose, attempted suicide or murder, drug-facilitated sexual assault, as well as using THZ eye drops to produce false negative urine illicit drug tests were included in this study.

### Exclusion criteria

Non-English articles, gray articles in the form of conference papers or abstracts, or any article that was not associated with non-medicinal or criminal uses of THZ was excluded. Articles that mentioned the use of other imidazole derivatives were excluded from this study. Studies that mentioned adverse effects of THZ or other similar substances in the context of “medicinal use purposes” was not included as our main aim was to identify its “off-label” or criminal uses.

### Outcomes

Depending on the type of study included, certain outcomes were investigated. If the study was a case report, documented outcomes included patient demographics (age and gender), amount of THZ ingested (serum concentration or the amount in milliliters ingested from a bottle), the clinical features of the patient upon admission to the hospital/ emergency department, the type of non-medicinal/criminal use it was intended for, and general case details about how, where, or when the event took place. If a study was a review or original article, general information about the study was recorded such as the objective of the study and the results of the study.

## Results

A total of 15 studies matched our inclusion criteria. Of the 15 studies included, 12 were case reports (which reported on a total of 18 cases), two articles were original research, and one article was a “letter to the editor” [4] but was originally discussing a case report and therefore included in the case report section of this study (Fig. 1).

### Original research results

From the search engines, there were only two original studies that matched our inclusion criteria [10, 11]. Both studies discussed the use of THZ containing eyedrops (specifically Visine ^©^) to falsify positive urine drug tests. The exact sample size for each study was not indicated, neither was the number of urine samples used to produce their results. The first study by Mikkelsen and Ash used eight different additives (suggested by drug users) and poured them into different types of positive urine drug tests. After analysis, they found that Visine altered the drug tests for urine samples containing benzodiazepines and marijuana (THC), but no change was noticed for sample that contained cocaine, barbiturates, opiates, and amphetamines [10]. Tests were confirmed using gas chromatography/mass spectrometry (GC/MS). The second study used a similar approach but only tested Visine eyedrops on a variety of urine samples containing illicit drugs. They found that THC-containing urine tested false negative after the addition of the eyedrops and noticed that the THZ-containing eyedrops did have a drug interference with other samples of urine like those containing oxazepam, benzoylecgonine, and phencyclidine. The interference with the eyedrops and these urine samples was affected but not to the point that the THZ eyedrops would decrease the cut-off value in the sample (the mechanism by which the sample presented as a false negative for THC), which is why the samples still came out positive for those substances [11]. Both studies concluded that THZ containing eyedrops can be used to falsify the results of some illicit drug tests. Both studies also pointed out that the eyedrops were undetectable in the sample, did not change the consistency or pH of the urine, and therefore concluded that it is an easy unidentifiable method to alter specific illicit drug test, especially cannabinoids.

### Case report results

Of the 18 case reports included in this study, two (11.1%) cases reported on drug-facilitated sexual assault/rape [9, 12], two (11.1%) cases used THZ eyedrops to attempt suicide, accidental ingestion of THZ eyedrops was reported in eight (44.4%) cases, three (16.7%) cases of attempted murder were reported, two (11.1%) cases of intentional ingestion were reported, and one (5.5%) case was a combination of drug-facilitated sexual assault (DFSA) and attempted murder (Table 1).

**Table 1:** Details regarding each case of THZ toxicity

Case:	Author:	Year:	G:	Age:	Clinical picture:	Amount of THZ:	Non-medicinal criminal use:	Case details:
1	Spiller et al.	2012	F	19Y	Lightheaded, groggy, brief periods of loss of consciousness followed by consciousness, spontaneous multiple episodes of vomiting, and mild bradycardia	Initially unknown; serum concentration was 108 ng/ml 24 h after ingestion	DFSA	Victim was invited to male friend’s house where he gave her a “special drink” for the evening. The victim experienced symptom and became unconscious. The male friend sexually assaulted, raped, and sodomized her. The next morning the patient was taken to the ER by a friend
2	Spiller et al.	2012	F	31Y	Initial tachycardia, loss of consciousness, and post-exposure amnesia, hypotension, pinpoint pupils, and burning sensation on the face	Unknown	DFSA and attempted murder	The victim was locked in her home and was threatened to inject herself with a mixture of illicit drug and THZ by her male partner. The male partner began pouring on her face this mixture as well until she lost consciousness and was then raped and sodomized
3	Stillwell and Saady	2012	F	16Y	Bradycardia, hypotension, dizziness, and loss of consciousness	Initially unknown, 7 h after ingestion the patient’s serum levels were 1.481 ng/ml	DFSA	Eyewitness noticed that male partner had a bottle of Visine^©^ eyedrops which was poured into the victim’s drink at a party
4	Spiller and Griffith	2007	F	17Y	Lethargy, slowed speech, ataxia, dizziness, orthostatic hypotension, and severe bradycardia (symptoms lasted 36 h)	2–3 ml was ingested	Incidental ingestion	Patient assumed that the bottle contained a “cough remedy” for her current upper respiratory tract infection
5	Suad et al.	2014	M	18 m	Diarrhea, vomiting, somnolence, hypotension, refractory bradycardia, and first-degree heart block	2430 ng/ml in urine and 21.56 ng/ml in serum (approximately 15 ml of THZ solution was ingested)	Attempted murder	The infant drank milk adulterated with THZ eye drops which was intended for a different family member
6	Lev and Clark	1995	M	41Y	Dry mouth, shortness of breath, dizziness, sinus bradycardia, severe hypotension	2 bottles of THZ containing eyedrops (30 ml)	Attempted suicide	Patient had asked advice from friends and bartenders on a quick and easy method to commit suicide, and they suggested drinking THZ-containing eyedrop bottles
7	Lowry et al.	2011	M	2Y	Lethargy, loss of consciousness, bradycardia, hypothermia, hypotension, and Cheyne-stokes respirations	51.4 ng/ml in serum	Incidental ingestion	The aunt had left her bottle of eyedrops at home that day, and it was suggested that the child had possibly played with the bottle accidentally ingesting it
8	Lowry et al.	2011	F	2Y	Lethargy, bradycardia, decreased respirations, and hypotension	39.3 ng/ml in serum, approximately 7.5 ml of the 15 ml bottle	Incidental ingestion	The child was found chewing on a bottle of Visine^©^ eyedrops
9	Lowry et al.	2011	M	20 m	Not arousable, pupil constriction, and CNS depression (which required mechanical ventilation)	24 ng/ml in serum, 0.30 mg/kg ingested	Incidental ingestion	The father found the child holding a bottle of Visine^©^ eyedrops
10	Lasala et al.	2014	M	3Y	Lethargy, bradycardia, hypotension, pinpoint pupil	Unknown	Intentional ingestion	The child was admitted to the emergency department with the mentioned symptoms and discharged after 3 days. 12 days after, the child was readmitted to the hospital with the same clinical picture. Evaluation of the mother revealed that she had Munchausen by proxy syndrome and was intentionally making her child sick
11	Gussow Leon	2020	F	32Y	Episodes of unexplained bradycardia and hypotension for a duration of 2 years, patient eventually died	Unknown	Murder	The spouse was intentionally poisoning his wife with THZ-containing eyedrops, which eventually lead to her death, and the man was charged with manslaughter
12	Gussow Leon	2020	M	64 Y	Unknown	Unknown amount of THZ ingested for a duration of 3 days	Murder	Wife pleaded guilty to manslaughter and confessed to killing her husband using THZ containing eyedrops. Wife was sentenced to 25 years in prison
13	Holmes et al.	1999	M	36 m	Increased lethargy, reduced muscle tone, and CNS depression which required mechanical ventilation	30 ml THZ containing eyedrops	Incidental ingestion	Unknown details regarding how infant obtained eyedrop bottle
14	Afify et al.	2021	M	76Y	A-V block, QT prolongation, bradycardia, and hypotension	Eight bottles of Visine^©^ eyedrops bottles (120 ml)	Attempted suicide	–
15	Osterhoudt and Henretig	2004	M	16Y	Dizziness, fatigue, bradycardia, chest pain, visual disturbances, and SAN arrest	Unknown	Intentional ingestion	At school, few students decided to play a prank on a student (victim) by adding a few drops of THZ containing eyedrops in his drink
16	Paksu et al.	2012	M	1Y	Hypotension, bradycardia, loss of consciousness, and hypothermia	Half a bottle of THZ containing nasal decongestant drops (approx. 15 ml)	Incidental Ingestion	One-year-old boy ingested half a bottle of THZ containing nasal decongestant drops. Other case details unknown
17	Tobias	1996	M	2Y	Hypotension, bradycardia, hypoglycemia, and progressive CNS depression requiring mechanical ventilation	2–3 ml	Incidental ingestion	Child was found playing with a THZ containing eyedrops bottle
18	Jensen et al.	1989	F	17 m	Bradycardia, hypotension, increasing lethargy, and somnolence	Approximately 1/3 of the bottle was ingested	Incidental ingestion	Child was seen playing with a bottle of THZ containing eyedrops

*G* gender, *M* male, *F* female, *Y* years, *m* months, *DFSA* Drug-facilitated sexual assault, *THZ* tetrahydrozoline

#### Case demographics

38.8% of cases were female victims (*n* = 7), and the remaining 61.1% were male victims (*n* = 11). 27.7% (*n* = 5) of the victims were from the adult/elderly age group (the youngest adult was 32, and the oldest was 76 years old). The remaining 72.2% of cases (*n* = 13) were from the infant till adolescent age group with the youngest being 18 months old and the oldest being 19 years old.

#### Signs, symptomatology, and method of administration

The most common presenting symptoms upon admission to the emergency department was severe bradycardia (*n* = 14, 77.7% of cases), severe refractory hypotension (*n* = 12, 66.6%), and loss of consciousness (*n* = 5, 27.7%). Severe clinical symptomatology was depicted in the younger age groups (infant to adolescent) in the form of ataxia, complete CNS depression which required mechanical ventilation, Cheyne-stokes respirations, and first-degree A-V block [2–4, 13]. Signs and symptoms of toxicity presented at various hours after ingestion, with symptoms appearing as early as three hours after ingestion and remaining up to 38 h after ingestion [1].

94.4% of cases (*n* = 17) orally ingested THZ containing eyedrops/nasal decongestive drops whether directly from the bottle or indirectly through a drink. One case was given THZ eyedrops in the form of “forced” intravenous injections.

#### Diagnostic evaluation

Of the 18 case reports, 11 (61.1%) were unable to identify the initial amount of THZ ingested, and it was only upon hospital admission were they able to figure out if a certain number of milliliters was missing from the bottle or determine values in serum or urine. The remaining seven (38.9%) cases knew the exact or a very close approximation to the amount of THZ eyedrops/nasal decongestant that was ingested.

Two out of 18 (11.1%) cases tested positive for illicit drugs on routine toxicology laboratory screening, three (16.6%) of the 18 cases did not present any data on routine toxicology screening results, one case did not perform routine toxicology screening since they already knew from the presenting history that the patient ingested tetrahydrozoline [14], and the remaining 12 (66.7%) cases had unremarkable routine toxicology screening. Only 10 cases reported on the serum concentration values of THZ using GC/MS, with the highest value being 114 ng/ml and the lowest concentration being 1.48 ng/ml (mean serum concentration was 49.14 ng/ml for the cases that reported values). From these 10 cases, it was noticed that the THZ concentration values were obtained at varying hours after hospital admission, with three hours being the earliest time that THZ presented in the serum and was noticed up to 24 h post ingestion (the mean hour of THZ serum detection was 12.2 h).

**Fig. 2:**
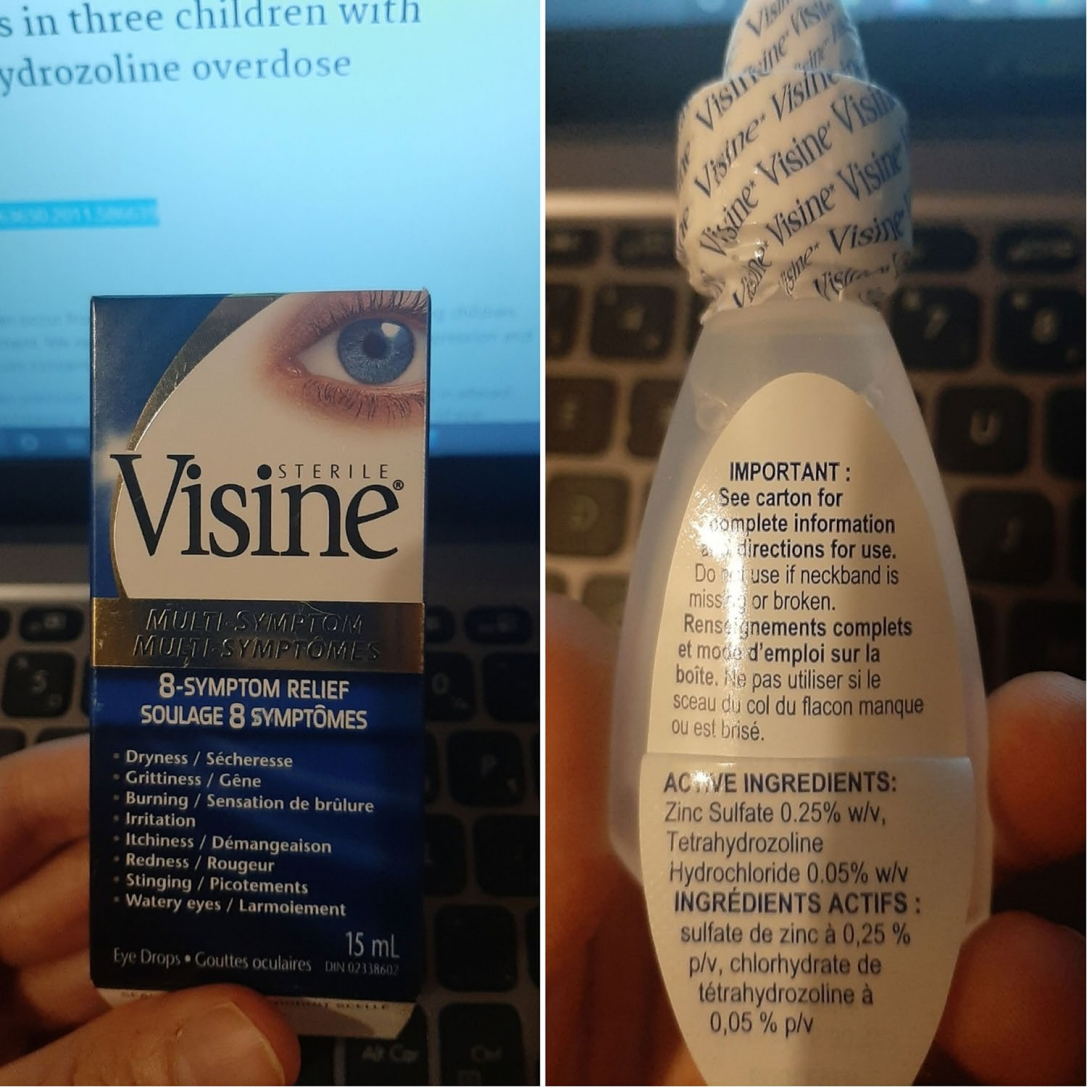
Visine eyedrops where the most commonly used THZ containing drops in the cases examined. No clear warning label exists on the bottle. Figure depicts a Visine bottle purchased OTC from Ontario, Canada

The only diagnostic method used to analyze the amount and presence of THZ toxicity was gas chromatography/mass spectroscopy (GC/MS) [5, 7, 15]. This was used in 100% of the cases and demonstrated positive results in all cases. Four (22.2%) of the cases were unable to identify the brand or name of eyedrops/nasal decongestant ingested, whereas 77.8% (*n* = 14) of cases mentioned that the type of THZ containing eyedrops ingested was from the brand Visine^©^ Tetrahydrozoline drops 0.05% (see Fig. 2) [5, 12].

#### Treatment modalities

Only 11 out of 18 case reports mentioned an approach to treating THZ toxicity. The remaining case reports did not mention the treatments used in their cases. There were similar treatment approaches taken by each hospital. Five of the ten cases used intravenous infusion of normal saline (NaCl 0.9%) to maintain blood pressure (since severe hypotension was a common presenting sign), three cases used 1–2 mg of naloxone [16], two cases used atropine (0.02–0.5 mg) [6, 14], and three cases used activated charcoal which was either administered orally or using a nasogastric tube. Two cases required mechanical ventilation, and one case was given supplemental oxygen using a nasal canula. All cases underwent observation for a various number of hours, with the longest observation time to be 72 h (three whole days) (Table 2).

**Table 2:** Treatment approaches of each reported case

Author:	Case:	Treatment:
Spiller et al.	1	No specific treatment mentioned, observation
Spiller et al.	2	No specific treatment mentioned, observation
Stillwell and Saady	3	No specific treatment mentioned, observation
Stillwell and Saady	4	No specific treatment mentioned, observation
Spiller and Griffith	5	IV infusion of 100 ml/hour of 0.9% NaCl, observation
Al-Abri et al.	6	IV 1 mg of Naloxone (no response to treatment), observation
Jensen et al.	7	10 mg activated charcoal (nasogastric tube) + IV normal saline
Lev and Clark	8	IV Atropine (at 3 different interval and doses – 0.5 mg, 1.0 mg, 3 mg) + 2 mg naloxone, 500 ml bolus of normal saline, observation
Tobias	9	2 mg naloxone, 1 ml/kg of 25% glucose (treat hypoglycemia), + mechanical ventilation, observation
Lowry and Garg	10	IV normal saline, observation
Lowry and Garg	11	Observation
Lowry and Garg	12	Mechanical ventilation, observation
Lasala et al.	13	No specific treatment mentioned, observation
Osterhoudt and Henretig	14	Supplemental oxygen (nasal canula) + 50 g activated charcoal mixed with sorbitol, observation
Paksu et al.	15	0.1 mg/kg naloxone + 0.02 mg/kg atropine, observation
Afify et al.	16	No specific treatment mentioned, observation

**Fig. 3:**
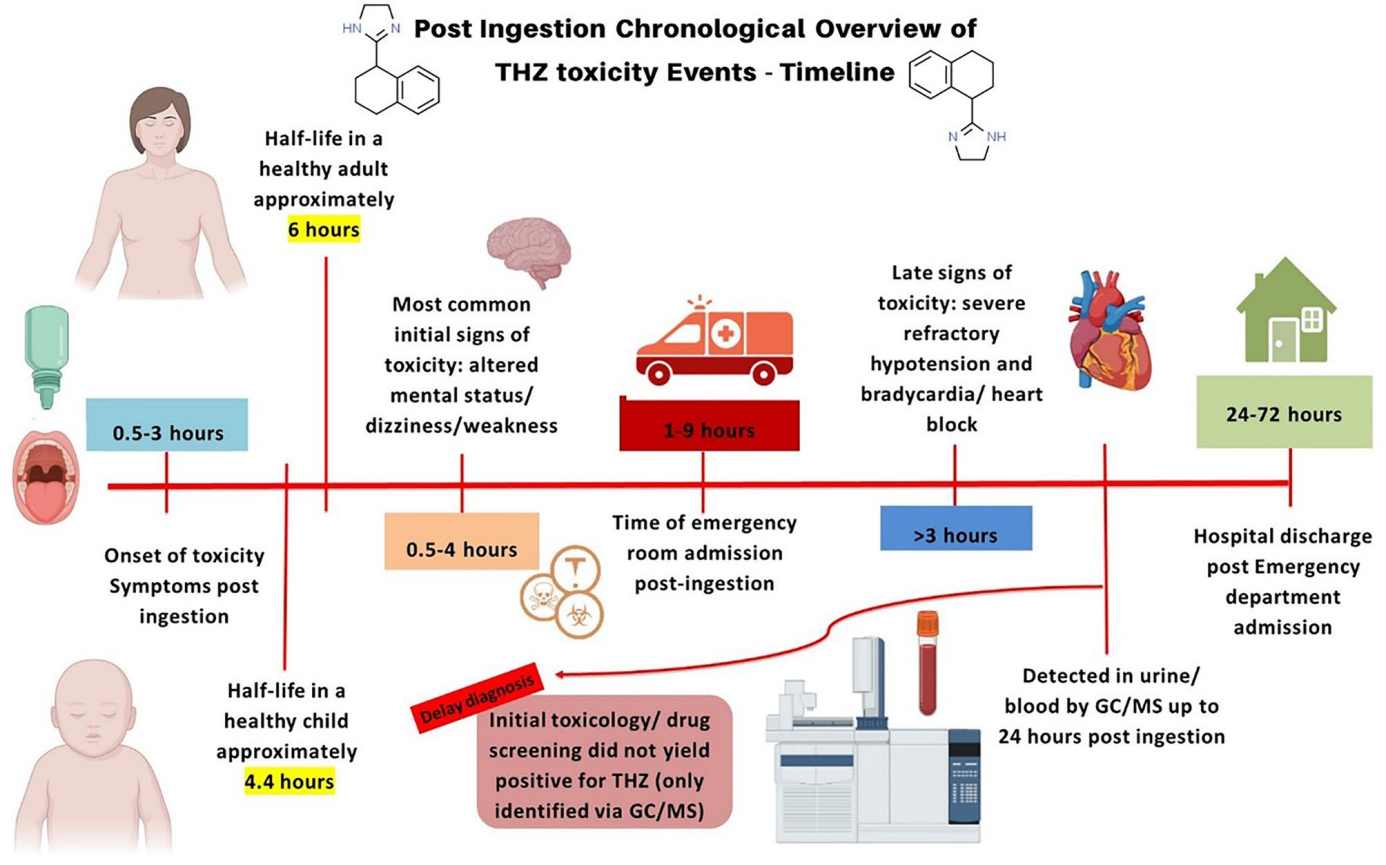
Post ingestion chronological overview of THZ toxicity events—timeline

It is important to note that any conclusion drawn by such results is simply reflective on the number of studies or cases that reported on the effect of THZ toxicity. Currently, there is limited information on tetrahydrozoline including the volume of distribution upon administration, its metabolism, the concentration levels at which it produces toxic effects depending on age group, its interaction with other medication, possible antidotes for its toxicity or overdose, and mechanisms of action based on method of administration. Figure 3 better depicts the chronological order of events that occur following the oral ingestion of THZ. This timeline is based on the information gathered from the included studies. Gaps or variations for certain events within the timeline (ex. time of hospital admission) may be attributed to the fact that THZ toxicity is not recognizable or easily suspected in emergency cases. Diagnosis is rarely suspected, and most commonly, upon admission, the clinical picture may be confused for illicit drug overdose. It should be noted that the nefarious uses of THZ are also rarely expected and reported on, which makes diagnosing and treating is that much harder when it comes to something like intentional overdoses. For these reasons, more studies are needed to fill in the voids on what is known about THZ.

## Discussion

### What we know about THZ

A study by Carr et al. successfully identified the baseline therapeutic serum and urine concentrations of tetrahydrozoline in healthy volunteers [7]. Their results revealed that when used as directed by the manufacturer, tetrahydrozoline is detectable in serum and urine up to 12 h. They suggested that a THZ concentration greater than the 95% confidence interval is most probably inappropriate use (adulterant, accidental, or suicidal), or in other words, if the level of THZ exceeds the upper limit (13–210 ng/ml in blood or 11–40 ng/ml in urine) is suggestive of non-medicinal use and warrants further investigation [7]. According to Tobias et al., the dosage necessary to produce a comatose state in child is 2–5 ml of 0.05% tetrahydrozoline solution, the same concentration found in Visine^©^ eyedrops (the most used brand in all the cases mentioned before) [3]. Spiller et al. pointed out that the lipophilic properties of THZ do not produce systemic side effects when given topically in the eye, but only when ingested orally [1, 9].

The results of this study suggest the use of THZ-containing eye/nasal drops in homicide, suicide, as a date rape drug, and interference of drug tests. There are several reasons why tetrahydrozoline can be used as a DFSA agent. In DFSA, the main intention is to produce a state of the victim where they cannot fend off their attacker. The primary drugs therefore being used include alcohol and benzodiazepines (one of the drugs that when mixed with THZ produces a false negative urine drug test) [9–12]. Other factors that are considered are drugs that cause memory impairment to prevent the victim from recalling details of the event, to impair judgment, to produce unconsciousness, and a drug that is not easily detectable on routine screens. The availability of the drug to the perpetrator is another factor. Tetrahydrozoline can be very easily obtained as it is available in over-the-counter eye drops and nasal decongestants; it is a colorless, odorless, and tasteless liquid. Purchase of such a bottle is also unlikely to arouse suspicion by either the victim or investigators. This was demonstrated by Stillwell et al., where the only proof of how THZ entered the victim’s system was eyewitness of customers at a bar noticing the male partner pouring Visine^©^ eyedrops into the victim’s drink, which he later identified as eyedrops he purchased because he had “red eye” [12]. THZ-containing drops can easily thwart the suspicion of victims or witnesses.

An interesting point that should be noted is its use for suicidal attempts. In the case reported by Lev et al., the 41-year-old male asked friends at a bar for “an easy way to commit suicide” and was recommended ingesting Visine THZ eyedrops. THZ containing drops may be used by bartenders and prostitutes use on aggressive or rowdy clients [6, 13]. Cases involving the nefarious use of THZ maybe higher than the actual reported cases as some victims may go unnoticed.

### Gaps in medical research

There is still much information to be investigated on THZ and its non-medicinal and criminal uses. More studies should be conducted to understand the mechanism of THZ toxicity (kinetics and dynamics) when ingested orally or otherwise, as well as studies on the values of toxicity or overdose, and clinical picture depending on different age groups. Treatment protocols for THZ and other imidazole derivatives should be developed. Mechanisms of absorption depending on mode of administration should be determined and interactions between THZ and other medications/drugs/and other chemicals should be studied. More studies should analyze alternative diagnostic methods and tests that are rapid and easy to use to help in quick diagnosis and prevent delay in treatment. More research should be made to see if there are different medication options that provide the same therapeutic effects without having such fatal effects when given in greater doses (few additional drops of THZ have proven to be fatal) [5, 14, 15].

### Gaps in forensic/legal framework

THZ is not a commonly suspected substance used in criminal activity, since its characteristics make it go unnoticed. The few cases mentioned in this study have pinpointed its non-medicinal criminal potential. More analysis should be made to determine if there are potential unreported criminal cases of THZ toxicity. Any case of child ingestion should be reported, especially in the case of suspected intentional ingestion, which was demonstrated in the case reported by Lasala et al., where the mother was intentionally feeding her child adulterated milk containing THZ eyedrops, which was associated with the mother having Munchausen by proxy syndrome [15]. Cases of DFSA should be investigated, as there may be a possibility that THZ was used (as demonstrated by three cases), especially since it is not commonly suspected in criminal activity. Cases of murder or “unexpected death” that involves the patient dying from unexplained bradycardia and hypotension should be analyzed for possible toxicity caused by imidazole derivatives like THZ (ex. post-mortem testing for THZ in serum) [8, 17] especially if the culprit has medical background. More studies should be done on what other illicit drug tests can be falsified using THZ containing medication, and mechanisms on how to detect altered toxicology laboratory testing may be developed. There is room for further development of tests that look for THZ as current screens do not consider it, as well as further investigations into ways to detect chronic poisoning by THZ.

### Further recommendations

Future research on the gaps of information mentioned above will clarify many unknowns about THZ. Purchasing of THZ containing drops may need to be monitored. Any case of ingestion associated with non-medicinal levels should be treated with suspicion. Routine toxicology screening should include THZ and other imidazole derivatives since it is typically misdiagnosed or misinterpreted for other illicit drugs (as demonstrated by its clinical picture). Healthcare providers should be proactively trained in awareness about THZ toxicity, as well as devise treatment protocols on how to manage these cases with their associated resistant symptoms. More tests should be made to easily detect its presence in blood and urine since some health facilities may not have the testing method for determining THZ values in system (GC/MS). Lastly, not all cases of child ingestion are accidental; further investigation may reveal intentional motives.

## Conclusion

This scoping review offers information regarding the non-medicinal criminal or “off-label” uses of THZ drops (eye/nasal) and has revealed gaps in the research that are still needed to be filled. From a medical standpoint, toxicity levels and side effects are not fully understood, which can be seen by the fact that different age groups had different levels of THZ in their body, each of which presented with different symptoms. The only commonality between them was severe refractory bradycardia and hypotension. In almost all cases, the method of treatment was observation and supportive care. Testing for THZ levels in blood and urine was only determined using gas chromatography/mass spectrometry, which is more commonly used for forensic laboratory investigations. Improved awareness and training in the healthcare field is needed to help better understand, diagnose, and treat THZ toxicity. Monitoring the purchases of THZ drops and applying warning labels on the bottles is increasingly necessary. THZ is frequently used as a DFSA drug; it should be considered in investigations and screened for in victims that report to the hospital. This review has visualized the need for more novel research and further investigations into cases of potential THZ intoxication.

## Key points

1. Tetrahydrozoline is a decongestant that can be used for non-medicinal criminal purposes.
2. The most common clinical features of excessive THZ ingestion include severe hypotension and bradycardia refractory to therapy.
3. Training healthcare workers, pharmaceuticals, and law enforcement on THZ toxicity should be done to aid in the identification and prevention of overdose or incidental ingestion.
4. Nefarious “off-label” uses of THZ include intentional poisoning, drug-facilitated sexual assault, murder, and suicide.

## Data Availability

The data based on this study is derived from the studies and their details listed in the tables. Any further details may be asked of the corresponding author (RM) with reasonable request.
